# Assessment of Acute and Chronic Toxicity in *Wistar* Rats (*Rattus norvegicus*) and New Zealand Rabbits *(Oryctolagus cuniculus)* of an Enriched Polyphenol Extract Obtained from *Caesalpinia spinosa*

**DOI:** 10.1155/2024/3769933

**Published:** 2024-04-10

**Authors:** Ricardo Ballesteros-Ramírez, Paola Lasso, Claudia Urueña, Jenny Saturno, Susana Fiorentino

**Affiliations:** ^1^Grupo de Inmunobiología y Biología Celular, Facultad de Ciencias, Pontificia Universidad Javeriana, Bogotá, Colombia; ^2^Grupo de Investigación de Fitoquímica, Facultad de Ciencias, Pontificia Universidad Javeriana, Bogotá, Colombia

## Abstract

Although herbal drugs are often considered safe for consumption, there is increasing evidence that some can generate undesirable health effects. However, polyphenols found in certain plants have been shown to provide a range of benefits for human health. In previous work, a standardized and quantified extract (P2Et) obtained from *Caesalpinia spinosa* (Dividivi) plant showed promising antioxidant, immunomodulatory, and anti-inflammatory properties in animal models of cancer and COVID-19 patients. The extract has also been subjected to genotoxicity, mutagenicity, and 28-day oral chronic toxicity evaluations, demonstrating a good safety profile. To advance preclinical and clinical development, further acute and chronic toxicity evaluations of the P2Et extract were performed. Acute toxicity tests were performed orally in *Wistar* rats at a dose of 2000 mg/kg, indicating that the lethal dose 50% (LD_50_) value exceeded 2000 mg/kg and classifying the P2Et extract as category 5 according to the Globally Harmonized System of Classification (GHS). In this work, chronic toxicity tests were conducted for 180 days on *Wistar* rats and *New Zealand* rabbits at a dose of 1000 mg/kg under Good Laboratory Practice (GLP) conditions. No weight loss or alterations in biochemical and hematological parameters associated with treatment were observed in the animals, suggesting the absence of toxicity in the assessed parameters. These results indicate that the P2Et extract is safe for oral administration at doses up to 1000 mg/kg body weight over a six-month period.

## 1. Introduction

Polyphenols are a group of naturally occurring compounds that have drawn significant interest in recent years due to their potential health benefits. Extensive research has established their diverse properties, including antitumor, antidiabetic, anti-inflammatory, antioxidant, and immunomodulatory effects, which make them an attractive target for therapeutic applications [1–4]. As a result, the development of polyphenol-enriched foods, dietary supplements, and phytomedicines has become an area of active research and development. The aim of offering individuals' safe and effective options for improving their health and well-being. The growing body of evidence on polyphenols supports the notion that these compounds could provide significant health benefits to individuals in the coming years [5].

However, one of the major challenges associated with herbal products is ensuring their quality and safety for human consumption. Chemical and biological variability of herbal drugs can affect their efficacy and safety, and toxicological support is needed to assess their safety profiles in chronic consumption [6]. It has been reported that certain polyphenols can have toxic effects when ingested in large bolus doses in a dose-dependent manner [7]. This highlights the importance of conducting comprehensive toxicological evaluations for polyphenol-enriched products. The FDA's (Food and Drug Administration) herbal drug guidance recommends that a complete toxicological profile should be established for herbal products based on the intended duration and frequency of use, ranging from acute toxicity, genotoxicity, mutagenicity to chronic toxicity [8].

*Caesalpinia spinosa*, a shrubby legume mainly found in northern South America, has been traditionally used for its medicinal properties, which include antiseptic, antidiarrheal, antifungal, astringent, antiscorbutic, antidysentery [9], and wound-healing effects [10], among others. The plant's pods are an abundant source of tannins [11]. A characterized, standardized, and quantified extract of *Caesalpinia spinosa* called P2Et was obtained previously [12]. The P2Et shows a high antioxidant [12], immunomodulatory, and antitumor capacity [13–17].

The safety of P2Et extract has been investigated through preclinical studies, including genotoxicity and mutagenicity assays [18]. In addition, subchronic oral toxicity studies were conducted in rats and rabbits according to international guidelines. The results of the 28-day toxicity study showed no significant toxicity, as evidenced by the absence of clinical, hematological, and biochemical changes, as well as macroscopic findings at necropsy [19]. These findings are consistent with other reports on polyphenols, either as isolated compounds or as part of complex extracts, which have shown low oral toxicity and no mutagenic or genotoxic effects [20–24].

In a four-week administration of P2Et to healthy volunteers, the maximum tolerated dose was determined to be 600 mg/daily [19]. These safety findings in humans are also supported by a multicenter randomized phase II clinical trial in hospitalized patients with COVID-19, where the administration of 500 mg daily of P2Et for 14 days showed no serious adverse events [25]. In Phase I clinical studies, polyphenols derived from gallic acid have been shown to be safe even when administered in increasing doses. This evidence further underscores the safety of extracts containing gallic acid polyphenols [26].

To complete the safety profile of P2Et, this study evaluated the acute and chronic toxicity of the extract for 14 and 180 days, respectively, in preclinical models. The LD_50_ value was determined using the guidelines set by The Organization for Economic Cooperation and Development (OECD) and International Council for Harmonisation of Technical Requirements for Pharmaceuticals for Human Use (ICH) [28]. This study also contributes to the broader understanding of the safety profile of polyphenols and herbal drugs, which may have important implications for the development of natural products for therapeutic purposes.

## 2. Materials and Methods

### 2.1. P2Et Extract

The P2Et extract was previously produced and standardized [12, 18], ensuring the consistency of both chemical and biological properties between batches, in compliance with FDA regulations [8]. The drug extraction ratio (DER) was 20-50:1, where the P2Et contains a high concentration of hydrolyzable tannins ranging from 70 to 95%. These tannins are present in the form of mono-, di-, and tri-O-galloyl quinic derivatives, with a calculated amount of 5–30% gallic acid and 2–7% methyl gallate and ethyl gallate. The P2Et extract was produced under Good Manufacturing Practices (GMP) conditions. Furthermore, prior to any experiments, the extract was subjected to physicochemical and microbiological certification to ensure consistency of quality.

### 2.2. Toxicity Assessment

The toxicity evaluation was conducted in MedLab under Good Laboratory Practice (GLP) conditions. Animals were maintained in the test facilities according to local and international requirements, based on the Guide for the Care and Use of Laboratory Animals [27]. Animals that exhibited persistent signs of severe distress or pain were euthanized. These signs included abnormal vocalization, heightened aggressiveness, unusual posture, atypical reactions to handling, irregular movements, self-inflicted injuries, open wounds or skin ulcerations, respiratory difficulties, and corneal ulcerations. Other indicators for euthanasia included bone fractures, reluctance to move, unusual external appearance, rapid weight loss, emaciation, severe dehydration, significant bleeding, or any other symptoms, suggesting the animal might be in pain or distress during any test stage. Procedures for animal care and criteria for making the decision to euthanize an animal were based on the Guidance Document on the Recognition, Assessment, and Use of Clinical Signs as Humane Endpoints for Experimental Animals Used in Safety Evaluation [28]. The IACUC approval of the study is under the code FM-CIE-004-19.

#### 2.2.1. Acute Oral Toxicity

**Figure 1:**
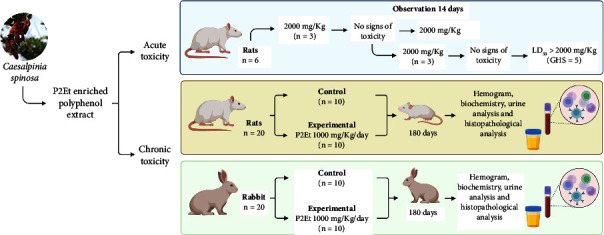
Schematic representation of the experimental design used to assess the acute and chronic oral toxicity of the P2Et extract.

The study was conducted following the guidelines outlined in the OECD no 423, 2001 [29]. Six females, Rattus norvegicus (rats), aged 8–12 weeks were used (Figure 1), average weight (225.10 ± 20.13), and we chose females because they generally exhibit a slightly higher sensitivity to toxic effects. The sample was prepared by mixing 5 g in 20 mL of 0.9% sodium chloride solution to achieve a concentration of 250 mg/mL. The dose of the sample administered was 2000 mg/kg body weight (BW). The rats were housed in a specific room with a temperature range of 20.1 to 22.2°C, relative humidity of 70.2 to 81.8%, and photoperiod 12/12 hours and underwent an acclimation period of one week. Feeding of the animals was composed of conventional feed for the species and filtered drinking water. Individual identification of the animals was established by random selection and numbering using a hydrographic pen. The animals were fasted overnight prior to sample administration and for 3–4 hours after administration.

Their individual body weights were recorded on the day of administration (Day 0) and on Days 7 and 14. In a previous study [18], we identified the maximum tolerated dose to be 2000 mg/kg BW, leading us to choose this dose for our test. The sample was given orally as a one-time dose through an appropriate gastric cannula. We began by treating three females with the 2000 mg/kg BW dose. Observing no significant clinical signs or mortality, we proceeded to treat an additional three females under the same parameters.

The animals were individually observed for the first 30 minutes after administration and periodically during the first 24 hours, with particular attention paid during the first 4 hours and 14 days. Observations included monitoring changes in skin, eyes, and mucous membranes and in the nervous, respiratory, circulatory, somatomotor, and behavioral systems. Special attention was given to observing tremors, convulsions, salivation, diarrhea, lethargy, and coma. Necropsy was performed, and the macroscopic findings are described in the Results section.

#### 2.2.2. Chronic Toxicity Evaluation

The study was conducted in compliance with ICH M3 (R2) guidelines [30] at a GLP-certified center. A total of 20 male and female (10 male and 10 female) rats (*Rattus norvegicus*) at seven weeks, and 20 (10 male and 10 female) rabbits (*Oryctolagus cuniculus*) at 12 weeks were used for the evaluation, with ten animals distributed evenly between control and experimental groups (Figure 1). Animals in the test group were labeled from 1 to 10, while those in the control group were labeled from 11 to 20. Additionally, a “M” was used to denote male animals, and “F” indicated female animals. In rats, the average weight for the female test group was 195.33 ± 7.16, compared to 191.69 ± 5.49 for the control group (*p* value: 0.39). In male rats, the average weight of the test group was 316.52 ± 18.54, compared to 317.28 ± 18.85 in the control group (*p* value: 0.95). For rabbits, given that both genders exhibit comparable weights, the test group had an average weight of 2950.8 ± 227.0, while the control group's average stood at 3097.8 ± 300.3 (*p* value: 0.23).

The rats were housed in a specific room with a temperature range of 17.0–20.6°C and relative humidity between 54.9 and 70.2%, while rabbits were housed in a room with a temperature range of 17.4–20.8°C and relative humidity between 54.9 and 70.2%. Both species were subjected to a photoperiod consisting of 12 hours of light followed by 12 hours of darkness. Rats underwent an acclimation period of at least one week, while rabbits acclimated for at least two weeks.

The animals were fed conventional food for their respective species and provided with filtered drinking water.

Water and food intake were assessed through 24-hour measurements taken weekly throughout the experimental period for both rats and rabbits. Clinical toxicity signs and death were monitored, and a registry was maintained weekly. At the end of the 180 days, three distinct blood samples were collected for analysis: one for whole blood analysis, one for serum separation, and another for plasma obtaining. Hemograms were conducted on the whole blood sample, collected in EDTA tubes, using both automated and manual techniques. Coagulograms were performed on the plasma, which is obtained from blood collected in sodium citrate tubes, utilizing the thromboplastin (prothrombin time, PT) and ellagic acid (partial thromboplastin time, PTT) methods. Standard hematological internal techniques, practiced in the laboratory, were employed for the measurement of the parameters.

The biochemical were performed in serum, the blood samples were centrifuged at 5000 rpm for 15 minutes, and the serum was kept at −20°C until analysis for clinical biochemistry measurements. Standardized diagnostic kits by Analisa® and a Biotron® spectrophotometer were used in determination of the biochemical. Euthanasia was carried out on both rats and rabbits using an intraperitoneal injection of xylazine and ketamine at doses of 300 mg/kg BW and 30 mg/kg BW [31], respectively. Subsequently, the organs were weighed. The liver, left kidney, left adrenal, and spleen's absolute weight (AW) were documented (Semianalytical and Analytical Scale Ohaus Adventurer). The relative weight (RW) calculation was performed according to the formula: (Organ weight/Final body weight) × 100, in accordance with established internal laboratory procedures. Urine samples were also collected for analysis, and these samples were promptly refrigerated at 2–8°C, though we aim to analyze them immediately. The control group received the dilution vehicle in filtered water, with the same conditions applied to the P2Et group. Animal weights were recorded on Day 0 and weekly for six months. P2Et was administered orally once a day for six months at a dose of 1000 mg/kg using a syringe and gastric tube, with the administration volumes adjusted according to the animal's weight. For rats, urine analysis was performed using pooled samples from animals within the same group, as it was not possible to collect sufficient volumes of urine per individual animal.

#### 2.2.3. Statistical Analysis

The statistical analysis of toxicity tests was conducted using BioEstat 5.3 software and GraphPad Prism v9.3.1 for Mac OS X (GraphPad Software, La Jolla California United States, https://www.graphpad.com). For comparing the experimental group with the control group, Student's t test was utilized for parametric data, while the nonparametric Mann–Whitney test was employed for nonparametric data. To evaluate the normality of data in the chronic toxicity test, the Shapiro–Wilk test was performed, and the nonparametric Mann–Whitney test was applied for non-normal data. The level of significance used in the analysis was set at *p* < 0.05, indicating a statistically significant difference between groups.

## 3. Results

### 3.1. Acute Toxicity

**Figure 2:**
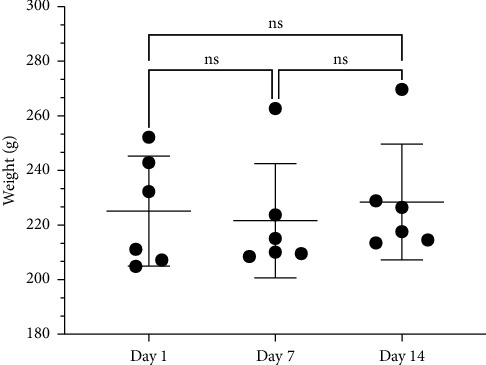
Weight variation during P2Et acute toxicity assessment.

During the observation period, two animals showed a decrease in body weight (Figure 2). However, the weight loss observed in these animals was not associated with any clinical signs of toxicity, and no macroscopic organ alterations were identified during necropsy. The remaining animals did not show any significant changes in their body weight or clinical symptoms throughout the study period.

Additionally, no relevant alterations were detected in the different physiological systems assessed during the necropsy phase, including the cardiovascular, respiratory, digestive, and nervous systems. According to the Globally Harmonized System of Classification and Labelling of Chemicals (GHS), the P2Et was classified as category 5, indicating a low level of toxicity, with an LD_50_ value greater than 2000 mg/kg.

### 3.2. Chronic Toxicity

The experimental period was conducted without any observed clinical signs of toxicity, indicating that the experimental conditions were well-tolerated by the animals. The comparison of initial weight, final weight, and body weight gain between males and females of both the experimental and control groups in rats and rabbits showed no statistical differences (Table 1). Moreover, water consumption did not differ significantly between the experimental and control groups during the investigation period, supporting the absence of any experimental interference (Table 1). Although there was a notable difference in feed consumption between the experimental and control groups among female rats in the second and sixth months, it did not significantly affect the overall study outcomes. Male rats demonstrated a notable variance in food consumption exclusively during the fourth month, while female rats showed this difference at the second and sixth months. However, these fluctuations were deemed to be of no consequence to the overall objectives of the study. Regarding the rabbits, a discernible difference in feed intake was apparent only in the fourth and fifth months. In the fourth month, the control group exhibited a higher consumption rate, whereas in the following month, the experimental group's intake surpassed that of the control group (Tables S5–S8).

In the male rat cohort, analysis revealed no statistically significant differences in both the absolute and relative weights of the liver, spleen, and kidneys when comparing the experimental group with the control group. Conversely, in the female rats, a notable difference was observed in both the absolute and relative weights of the adrenal glands, as detailed in Table 2. Urea levels showed a statistically significant difference between the experimental and control groups, with the control group exhibiting higher levels, and potassium levels showing the opposite pattern (Table 3). The evaluation of hemogram results (erythrogram and leukogram), coagulogram, liver function, and kidney function showed no significant systemic alterations in any of the animals (Table 4).

An animal (8M) from the test group exhibited diarrhea on the study's initial day but showed recovery in the following days. Four animals from the test group, specifically two females (2F and 3F) and two males (7M and 8M), succumbed during the experimental phase due to complications from incorrect substance administration (Table S1). However, no clinical signs of toxicity or fatalities attributable to the test substance's effects were noted. Notably, an animal (4F) from the test group had duodenal congestion. Meanwhile, two control group animals (17M and 19M) exhibited a thickened pancreas appearance, and another control group animal (20M) had nodules and vesicles present in the lungs (Table S2).

In the rabbits group, study commenced with a total of 20 animals, evenly split between the test and control groups, and balanced in terms of gender. However, early in the study, four animals succumbed due to complications from incorrect substance administration. To compensate, four additional animals were introduced. As the study progressed, five more animals either died or were euthanized due to similar administration issues (Table S3). Additionally, two animals were humanely euthanized because of a facial abscess and a spinal injury sustained while attempting to flee the cage. Consequently, the study concluded with three females and four males in the test group, and two females and three males in the control group (Table S3).  Table S4 shows the macroscopic evaluation at necropsy examination after the end of the experimental period. In the test group, animal 6M showed dilation of urinary bladder and strangulation in one border of spleen, animal 7M showed hemorrhagic areas and abscesses in the lung, and animal 11F presented edema and hemorrhagic areas in the lung. In the control group, animal 17M showed congestion in cecum and duodenum and pancreas with dark color; animal 20M showed congestion in cecum and duodenum; animal 12F presented lymph node and pancreas with dark color; and animal 15F presented pancreas with dark color and presence of small vesicle in the left adrenal. In general, all observations matched typical background abnormalities in healthy rabbits of the same age and type used in this research. These were seen as natural or random occurrences and were not connected to the P2Et treatment.

The only significant difference between the experimental and control groups was the partial thromboplastin time (PTT) test, which was higher in the experimental group (Table 5). However, all values were significantly below the reference values for the species in both groups, indicating that this alteration was not clinically relevant. Although atypical values were observed in some animals (high monocyte count in two animals in the experimental group, high aspartate aminotransferase (AST) value of one animal from the control group, low bilirubin of one animal from the experimental group, and two animals from the control group), these changes were not considered relevant to the study since they were isolated cases in the groups and were not associated with any clinical signs (Table 6). Additionally, some parameters were found outside the reference value ranges for the species, such as PTT and triglycerides, which may indicate natural variability between the evaluated animals and the literature values. Overall, the absence of significant systemic alterations in the animals indicated that the experimental conditions were well-controlled and that the results obtained were reliable.

## 4. Discussion

Plant extracts have become a promising source of natural compounds for the development of dietary supplements or herbal drugs, and their preclinical regulatory phases are being fulfilled [32, 33]. *Caesalpinia spinosa* is a plant that has attracted attention due to its rich content of polyphenolic compounds, which have been linked to various health benefits. P2Et is a standardized and quantified extract obtained from *Caesalpinia spinosa*, which has shown antioxidant, immunomodulatory, and antitumor activities in previous studies [3, 12, 13, 15–17]. In a previous study, we reported that P2Et is not mutagenic or teratogenic [18]; moreover, we found no significant changes in toxicity after administering repeated doses of P2Et for 28 days [19], suggesting that it is relatively safe for chronic use. However, before P2Et can be introduced into the market as a dietary supplement or herbal drug, its safety needs to be evaluated rigorously. To this end, the current study conducted both acute and chronic toxicity evaluations to determine the safety profile of P2Et.

After conducting acute toxicity tests, our findings indicate that P2Et falls under category 5 in the GHS, with a LD_50_ greater than 2000 mg/kg. Although we administered a single dose of 2000 mg/kg to assess acute toxicity, two rats experienced weight loss without exhibiting any other clinical signs or macroscopic abnormalities during autopsy. Interestingly, gallic acid, one of the primary constituents of P2Et, has also been found to have an LD_50_ exceeding 2000 mg/kg [34], a trait shared by other polyphenolic compound-rich extracts [32, 35].

During our chronic toxicity study, it was observed no significant deviations in clinical signs of toxicity or any symptoms pertinent to the objectives of the study between the experimental and control groups, with the exception of elevated potassium levels in rats and increased RBC counts in rabbits. The heightened potassium levels did not correspond with findings from previous studies involving other polyphenolic compounds. This unusual elevation prompts considerations of renal efficiency, as the kidneys are primarily responsible for potassium excretion. Initial hyperkalemia can manifest even when chloride, calcium, and sodium levels are within normal ranges, a scenario reminiscent of early kidney dysfunction. Such alterations necessitate vigilant monitoring and further research to discern potential implications for renal and cardiovascular health with P2Et extract. It is crucial to consider that the consequences of increased serum potassium may vary with the extent of hyperkalemia and are influenced by individual and species-specific factors. This variation underscores the need for enlarging the study sample size and potentially investigating any nephron-related anomalies in future studies, despite the absence of kidney damage in necropsies and other parameters assessed in this study.

Regarding the augmented RBC counts, it is recognized that polyphenols can stimulate erythropoiesis via several pathways. These pathways include boosting hemoglobin levels [36] and elevating *α*-tocopherol, a variant of vitamin E that plays a role in the genesis and function of red blood cells [37]. Additionally, polyphenols may augment the antioxidant defense of red blood cells by binding to their surfaces, thereby improving their oxidant-scavenging efficacy. Consequently, these mechanisms may collectively contribute to a secondary rise in RBC count by bolstering the blood's overall antioxidant capacity [38]. It is important to note that despite the extensive use of *Caesalpinia spinosa* in various traditional preparations [39], no chronic toxicity studies have been conducted to evaluate the safety of these preparations or standardized extracts. Interestingly, there are no reports of toxicity, either acute or chronic, associated with the use of any other species of the genus *Caesalpinia* [40, 41].

The results we obtained play a fundamental role in determining the NOAEL (No Observed Adverse Effect Level) for P2Et, set at 1000 mg/kg BW following 180 days of consistent administration. This NOAEL designation derives from the lack of adverse effects observed throughout our study. While we recognize that a dose as high as 1500 mg/kg might not have manifested adverse effects either, our previous human clinical trials, with daily doses of 600 mg and 500 mg, did not exceed a 10-fold exposure margin to clinical exposure, and the clinical dose remained below 1 g per day as recommended in the guideline to use 1000 mg/kg BW as selected dose. It is also noteworthy that the NOAEL values for other polyphenolic compounds, sourced from green tea, have been documented to be marginally lower than P2Et in earlier research [42]. These results suggest that P2Et is comparatively less toxic and may have a higher safety margin than other polyphenolic compounds found in green tea.

The results of our toxicity evaluations support the continued development of P2Et as a potential herbal therapeutic agent. Nevertheless, the safety data obtained in this study provide a solid foundation for the future development of P2Et as a natural product-based therapy in chronic use.

## 5. Conclusions

These findings provide a basis for the safe use of P2Et extract in potential clinical trials involving human subjects in chronic use. However, it is important to note that further research is needed to fully evaluate the safety of P2Et extract in human subjects. These results pave the way for future studies to explore the potential clinical applications of P2Et extract with confidence in its safety profile.

## Figures and Tables

**Table 1:** Mean intake of food/water as well as initial and final body weight in rats and rabbits following 180 days of administration.

Animal	Sex	Group	*N*	Initial weight (g)	Final weight (g)	Body weight gain^*∗*^ (g)	Food (g)	Water (mL)
Rats	Females	Test	5	195.33 ± 7.16	265.38 ± 17.17^*∗∗∗*^	68.12 ± 18.48^*∗∗∗*^	16.5 ± 3.9	29.2 ± 5.7
		Control	5	191.69 ± 5.49	251.54 ± 20.60	59.85 ± 20.22	15.3 ± 2.0	26.1 ± 12.2
		*p* value		0.39	0.37	0.59	0.49	0.64
	Males	Test	5	316.52 ± 18.54	545.90 ± 37.33^*∗∗∗*^	218.47 ± 30.56^*∗∗∗*^	24.0 ± 10.5	37.5 ± 4.2
		Control	5	317.28 ± 18.85	510.10 ± 38.26	192.82 ± 25.92	28.0 ± 3.3	37.5 ± 4.2
		*p* value		0.95	0.24	0.25	0.51	0.60

Rabbits	Females	Test	5	2998 ± 94.12	5027 ± 631.30^*∗∗∗*^	2029 ± 537.19^*∗∗∗*^	163.5 ± 68.9	248.8 ± 158.5
		Control	5	3258 ± 299.74	5848 ± 65.05^*∗∗*^	2590 ± 234.70^*∗∗*^	177 ± 33.9	260.0 ± 146.7
		*p* value		0.10	0.18	0.65	0.67	0.89
	Males	Test	5	2903 ± 318.58	4221 ± 419.53^*∗∗∗∗*^	1317.40 ± 100.95^*∗∗∗∗*^	180.6 ± 48.6	324.3 ± 165.9
		Control	5	2937 ± 221.26	4538 ± 292.40^*∗∗∗*^	1601.07 ± 71.14^*∗∗∗*^	140.8 ± 46.7	236.0 ± 137.0
		*p* value		0.85	0.32	0.73	0.19	0.35

*N*: number of animals in the group, data expressed as mean ± standard deviation. ^*∗*^Body weight gain = initial weight–final weight. Number of animals per group at the end of the experiment: ^*∗∗*^*N* = 2, ^*∗∗∗*^*N* = 3, ^*∗∗∗∗*^*N* = 4.

**Table 2:** Absolute and relative weight of liver, left kidney, left adrenal, and spleen in rats and rabbits.

	Sex	Group	*N*	Liver		Kidney L		Adrenal L		Spleen	
				AW (g)	RW (%)	AW (g)	RW (%)	AW (g)	RW (%)	AW (g)	RW (%)
Rats	Females	Test	3	9.31 ± 0.73	3.51 ± 0.08	0.99 ± 0.02	0.38 ± 0.02	0.0483 ± 0.0036	0.0182 ± 0.0012	0.56 ± 0.10	0.21 ± 0.02
		Control	5	8.24 ± 0.61	3.29 ± 0.31	0.91 ± 0.10	0.36 ± 0.04	0.0370 ± 0.042	0.0148 ± 0.0023	0.49 ± 0.10	0.19 ± 0.03
	*p* value			0.07	0.22	0.15	0.63	0.008	0.06	0.36	0.49
	Males	Test	3	16.63 ± 1.88	3.05 ± 0.29	1.55 ± 0.23	0.28 ± 0.03	0.0338 ± 0.0038	0.0062 ± 0.006	0.81 ± 0.12	0.15 ± 0.01
		Control	5	17.01 ± 1.61	3.35 ± 0.38	1.58 ± 0.07	0.31 ± 0.03	0.0336 ± 0.0083	0.0065 ± 0.0011	0.80 ± 0.12	0.16 ± 0.02
	*p* value			0.77	0.29	0.88	0.29	0.97	0.72	0.87	0.57

Rabbits	Test		7	84.13 ± 11.31	1.925 ± 0.357	9.66 ± 0.83	0.221 ± 0.031	0.256 ± 0.069	0.006 ± 0.002	2.31 ± 0.55	0.052 ± 0.010
	Control		5	91.16 ± 13.90	1.801 ± 0.088	10.85 ± 1.11	0.216 ± 0.015	0.302 ± 0.067	0.006 ± 0.001	2.43 ± 0.56	0.048 ± 0.010
	*p* value			0.36	0.41	0.06	0.74	0.28	0.8	0.71	0.52

**Table 3:** Effects of P2Et extract on biochemical parameters in rats following 180 days of administration.

	Parameters (reference values)	Group		*p* value
		Test (*N* = 6)	Control (*N* = 8)	
Kidney function	Urea (18.0 to 45.0 mg/dL)	47.30 ± 5.91	57.41 ± 6.29	0.01^*∗*^
	Creatinine (0.05 to 0.65 mg/dL)	0.40 ± 0.09	0.38 ± 0.05	0.54
	Sodium (135.0 to 146.0 mmol/L)	148.95 ± 8.82	153.99 ± 21.17	0.52
	Potassium (4.0 to 5.9 mmol/L)	7.95 ± 2.20	6.16 ± 0.77	0.05^*∗*^
	Chloride (96.0 to 107.0 mmol/L)	100.30 ± 1.11	99.76 ± 2.26	0.60
	Calcium (5.3 to 11.6 mg/dL)	10.97 ± 0.93	10.28 ± 0.59	0.12

Liver function	ALT (20 to 61 U/L)	83.00 ± 39.59	95.13 ± 30.98	0.12
	Phosphatase (16 to 302 U/L)	190.33 ± 55.32	249.75 ± 59.88	0.08
	AST (39.0 to 111.0 U/L)	199.17 ± 153.83	213.88 ± 55.98	0.16
	GGT (0 to 6 U/L)	5.00 ± 0.00	4.88 ± 0.35	0.70
	Bilirubin (0.1 to 0.7 mg/dL)	0.17 ± 0.18	0.10 ± 0.00	0.61
	Cholesterol (20 to 92 mg/dL)	53.00 ± 10.79	50.13 ± 6.06	0.70
	Triglycerides (27 to 108 mg/dL)	164.60 ± 48.70	130.29 ± 52.37	0.24

*N*: number of animals in the group, data expressed as mean ± standard deviation. ^*∗*^Statistically significant difference *p* < 0.05. ALT: alanine transaminase); AST: aspartate aminotransferase; GGT: gamma-glutamyl transferase.

**Table 4:** Effects of P2Et extract on hematological parameters in rats following 180 days of administration.

	Parameters (reference values)	Group		*p* value
		Test (*N* = 6)	Control (*N* = 8)	
Erythrogram	Erythrocyte (6.6 to 9.0 million/mm^3^)	6.62 ± 0.21	6.71 ± 0.52	0.69
	Hemoglobin (13.2 to 16.4 g/dL)	14.43 ± 0.51	14.08 ± 0.83	0.37
	Hematocrit (41.1 to 51.1%)	43.87 ± 2.12	43.25 ± 2.60	0.56
	MCV (52.5 to 65.4 *μ*3)	66.33 ± 4.32	64.54 ± 2.41	0.34
	MCH (16.5 to 21.3 pg)	21.83 ± 0.85	21.01 ± 0.93	0.12
	MCHC (30.2 to 34.5 g/dL)	32.93 ± 1.07	32.58 ± 0.84	0.49

Leucogram	Leukocytes (7.3 to 12.6 thousands/mm^3^)	5.52 ± 1.61	6.14 ± 1.33	0.44
	Neutrophil (28 to 44%)	39.17 ± 7.55	37.88 ± 3.94	0.68
	Eosinophil (1 to 5%)	0.67 ± 0.82	0.50 ± 0.76	0.70
	Lymphocytes (39 to 72%)	57.83 ± 7.36	59.13 ± 3.80	0.68
	Monocytes (3 to 12%)	2.33 ± 1.21	2.50 ± 1.20	0.80

Coagulogram	PT (seconds)	16.94 ± 1.50	16.14 ± 0.63	0.20
	Platelets (0.84 to 1.24 million/mm^3^)	0.73 ± 0.16	0.73 ± 0.14	0.99
	PPT (seconds)	21.16 ± 4.36	25.84 ± 4.51	0.09

Total protein	6.8 to 8.7 g/dL	7.00 ± 0.22	7.13 ± 0.38	0.49

*N*: number of animals in the group, data expressed as mean ± standard deviation. MCV: mean corpuscular volume; MCH: mean corpuscular hemoglobin; MCHC: mean corpuscular hemoglobin concentration (MCHC); PT: prothrombin time; PPT: partial thromboplastin time.

**Table 5:** Effects of P2Et extract on hematological parameters in rabbits following 180 days of administration.

	Parameters and reference values	Group		*p* value
		Test (*N* = 7)	Control (*N* = 5)	
Erythrogram	Erythrocytes (5.1 to 7.9 million/mm^3^)	6.07 ± 0.34	5.65 ± 0.39	0.08^*∗*^
	Hemoglobin (10.0 to 17.4 g/dL)	13.34 ± 0.71	12.24 ± 0.52	0.02
	Hematocrit (33.0 to 50.0%)	38.11 ± 2.24	36.18 ± 2.01	0.16
	MVC (57.8 to 66.5 *μ*3)	62.80 ± 0.99	64.24 ± 5.37	0.87
	MHC (17.1 to 23.5 pg)	21.99 ± 0.35	21.68 ± 1.06	0.49
	MCHC (29.0 to 37.0 g/dL)	35.03 ± 0.43	33.86 ± 1.47	0.10

Leucogram	Leukocytes (5.2 to 12.5 thousands/mm^3^)	7.34 ± 1.64	6.18 ± 2.34	0.33
	Neutrophil (20 to 75%)	56.29 ± 9.62	44.60 ± 8.62	0.06
	Eosinophil (1 to 4%)	0.43 ± 0.53	1.40 ± 1.52	0.19
	Lymphocytes (30 to 85%)	39.43 ± 9.36	52.40 ± 7.60	0.03
	Monocytes (1 to 4%)	3.86 ± 3.08	1.60 ± 0.89	0.11

Coagulogram	PT (7.2 to 7.8 seconds)	6.29 ± 0.26	6.72 ± 0.65	0.14
	Platelets (250 to 650 thousands/mm^3^)	382.71 ± 116.43	271.60 ± 36.79	0.05
	PTT (35 seconds)	19.29 ± 1.95	16.86 ± 0.65	0.02^*∗*^

Total protein	5.4 to 8.5 g/dL	6.11 ± 0.28	6.12 ± 0.44	0.38

*N*: number of animals in the group, data expressed as mean ± standard deviation. ^*∗*^Statistically significant difference *p* < 0.05. MCV: mean corpuscular volume; MCH: mean corpuscular hemoglobin; MCHC: mean corpuscular hemoglobin concentration (MCHC); PT: prothrombin time; PPT: partial thromboplastin time.

**Table 6:** Effects of P2Et extract on biochemical parameters in rabbits following 180 days of administration.

	Parameters and reference values	Group		*p* value
		Test (*N* = 7)	Control (*N* = 5)	
Kidney function	Urea (13.0 to 52.0 mg/dL)	51.37 ± 12.83	48.50 ± 6.76	0.66
	Creatinine (0.80 to 1.80 mg/dL)	1.28 ± 0.29	1.27 ± 0.15	0.91
	Sodium (138.0 to 148.0 mmol/L)	146.20 ± 2.13	146.30 ± 1.81	0.93
	Potassium (3.3 to 6.9 mmol/L)	4.84 ± 0.27	4.76 ± 0.17	0.55
	Chloride (92.0 to 112.0 mmol/L)	99.04 ± 2.84	101.36 ± 2.50	0.17
	Calcium (5.6 to 12.0 mg/dL)	12.17 ± 0.35	12.62 ± 0.74	0.19

Liver function	ALT (31 to 60 U/L)	81.43 ± 17.90	88.00 ± 26.23	0.62
	Phosphatase (90 to 145 U/L)	36.14 ± 5.58	45.40 ± 27.49	0.50
	AST (42.0 to 98.0 U/L)	28.86 ± 7.20	31.40 ± 11.93	0.65
	GGT (4 to 12 U/L)	5.86 ± 1.46	5.40 ± 0.55	0.81
	Bilirubin (0.30 to 0.80 mg/dL)	0.30 ± 0.08	0.23 ± 0.11	0.23
	Cholesterol (35 to 60 mg/dL)	45.00 ± 0.00	47.20 ± 3.90	0.26
	Triglycerides (124 to 156 mg/dL)	48.76 ± 5.59	55.48 ± 20.56	0.81

*N*: number of animals in the group, data expressed as mean ± standard deviation. ALT: alanine transaminase; AST: aspartate aminotransferase; GGT: gamma-glutamyl transferase.

## Data Availability

The data used to support the findings of this study are available from the corresponding author upon request.

## Supplementary Materials

Supplementary Materials Table S1: Weekly Clinical Evaluation of Test and Control Groups (Rats). Table S2: Necropsy Findings of Test and Control Groups (Rats). Table S3: Weekly Clinical Evaluation of Test and Control Groups (Rabbits). Table S4: Necropsy Findings of Test and Control Groups (Rabbits). Table S5: Mean Food Intake (g)—Rats. Table S6: Mean Water Intake (g)—Rats. Table S7: Mean Food Intake (g)—Rabbits. Table S7: Mean Food Intake (g)—Rabbits. Table S8: Mean Water Intake (g)—Rabbits.
