# Supplementary material for: Assessment of Acute and Chronic Toxicity in Wistar Rats (Rattus norvegicus) and New Zealand Rabbits (Oryctolagus cuniculus) of an Enriched Polyphenol Extract Obtained from Caesalpinia spinosa
Source: J Toxicol. 2024 Apr 10;2024:3769933. doi: 10.1155/2024/3769933 (PMC11023715; doi:10.1155/2024/3769933)
Supplement: Supplementary Materials — Table S1: Weekly Clinical Evaluation of Test and Control Groups (Rats). Table S2: Necropsy Findings of Test and Control Groups (Rats). Table S3: Weekly Clinical Evaluation of Test and Control Groups (Rabbits). Table S4: Necropsy Findings of Test and Control Groups (Rabbits). Table S5: Mean Food Intake (g)—Rats. Table S6: Mean Water Intake (g)—Rats. Table S7: Mean Food Intake (g)—Rabbits. Table S7: Mean Food Intake (g)—Rabbits. Table S8: Mean Water Intake (g)—Rabbits. [file 3769933.f1.docx]

## Table S1: Weekly clinical evaluation of test and control groups (rats)

| **Group** | **Animal** | **Day** | **Day** | **Day** | **Day** | **Day** | **Day** | **Day** | **Day** | **Day** | **Day** | **Day** | **Day** | **Day** | **Day** | **Day** | **Day** | **Day** | **Day** | **Day** | **Day** | **Day** | **Day** | **Day** | **Day** | **Day** | **Day** |
| --- | --- | --- | --- | --- | --- | --- | --- | --- | --- | --- | --- | --- | --- | --- | --- | --- | --- | --- | --- | --- | --- | --- | --- | --- | --- | --- | --- |
|  |  | **0** | **7** | **14** | **21** | **28** | **35** | **42** | **49** | **56** | **63** | **70** | **77** | **84** | **91** | **98** | **105** | **112** | **119** | **126** | **133** | **140** | **147** | **154** | **161** | **168** | **175** |
|  | **1F** | NO | NO | NO | NO | NO | NO | NO | NO | NO | NO | NO | NO | NO | NO | NO | NO | NO | NO | NO | NO | NO | NO | NO | NO | NO | NO |
|  | **2F** | NO | NO | NO | NO | NO | NO | NO | NO | NO | NO | NO | NO | NO | NO | NO | 1* | --- | --- | --- | --- | --- | --- | --- | --- | --- | --- |
|  | **3F** | NO | NO | NO | NO | NO | NO | 1* | --- | --- | --- | --- | --- | --- | --- | --- | --- | --- | --- | --- | --- | --- | --- | --- | --- | --- | --- |
|  | **4F** | NO | NO | NO | NO | NO | NO | NO | NO | NO | NO | NO | NO | NO | NO | NO | NO | NO | NO | NO | NO | NO | NO | NO | NO | NO | NO |
| **Test** | **5F** | NO | NO | NO | NO | NO | NO | NO | NO | NO | NO | NO | NO | NO | NO | NO | NO | NO | NO | NO | NO | NO | NO | NO | NO | NO | NO |
|  | **6M** | NO | NO | NO | NO | NO | NO | NO | NO | NO | NO | NO | NO | NO | NO | NO | NO | NO | NO | NO | NO | NO | NO | NO | NO | NO | NO |
|  | **7M** | NO | NO | NO | NO | NO | NO | NO | NO | NO | NO | NO | NO | 1* | --- | --- | --- | --- | --- | --- | --- | --- | --- | --- | --- | --- | --- |
|  | **8M** | 11 | NO | NO | NO | NO | NO | NO | NO | 1* | --- | --- | --- | --- | --- | --- | --- | --- | --- | --- | --- | --- | --- | --- | --- | --- | --- |
|  | **9M** | NO | NO | NO | NO | NO | NO | NO | NO | NO | NO | NO | NO | NO | NO | NO | NO | NO | NO | NO | NO | NO | NO | NO | NO | NO | NO |
|  | **10M** | NO | NO | NO | NO | NO | NO | NO | NO | NO | NO | NO | NO | NO | NO | NO | NO | NO | NO | NO | NO | NO | NO | NO | NO | NO | NO |
|  | **11F** | NO | NO | NO | NO | NO | NO | NO | NO | NO | NO | NO | NO | NO | NO | NO | NO | NO | NO | NO | NO | NO | NO | NO | NO | NO | NO |
|  | **12F** | NO | NO | NO | NO | NO | NO | NO | NO | NO | NO | NO | NO | NO | NO | NO | NO | NO | NO | NO | NO | NO | NO | NO | NO | NO | NO |
|  | **13F** | NO | NO | NO | NO | NO | NO | NO | NO | NO | NO | NO | NO | NO | NO | NO | NO | NO | NO | NO | NO | NO | NO | NO | NO | NO | NO |
|  | **14F** | NO | NO | NO | NO | NO | NO | NO | NO | NO | NO | NO | NO | NO | NO | NO | NO | NO | NO | NO | NO | NO | NO | NO | NO | NO | NO |
| **Control** | **15F** | NO | NO | NO | NO | NO | NO | NO | NO | NO | NO | NO | NO | NO | NO | NO | NO | NO | NO | NO | NO | NO | NO | NO | NO | NO | NO |
|  | **16M** | NO | NO | NO | NO | NO | NO | NO | NO | NO | NO | NO | NO | NO | NO | NO | NO | NO | NO | NO | NO | NO | NO | NO | NO | NO | NO |
|  | **17M** | NO | NO | NO | NO | NO | NO | NO | NO | NO | NO | NO | NO | NO | NO | NO | NO | NO | NO | NO | NO | NO | NO | NO | NO | NO | NO |
|  | **18M** | NO | NO | NO | NO | NO | NO | NO | NO | NO | NO | NO | NO | NO | NO | NO | NO | NO | NO | NO | NO | NO | NO | NO | NO | NO | NO |
|  | **19M** | NO | NO | NO | NO | NO | NO | NO | NO | NO | NO | NO | NO | NO | NO | NO | NO | NO | NO | NO | NO | NO | NO | NO | NO | NO | NO |
|  | **20M** | NO | NO | NO | NO | NO | NO | NO | NO | NO | NO | NO | NO | NO | NO | NO | NO | NO | NO | NO | NO | NO | NO | NO | NO | NO | NO |

F: female; M: male. Clinical alterations observed during the study ranged from no observations (NO) to various symptoms including death (1), convulsion (2), mutilation (3), prostration (4), ataxia (5), tremors (6), local inflammation (7), dyspnea (8), tearing (9), salivation (10), diarrhea (11), piloerection (12), and cachexia (13). * death by false route of administration

## Table S2: Necropsy Findings of Test and Control Groups (Rats)

| **Group** | **Animal** | **Macroscopic findings** |
| --- | --- | --- |
| **Test** | **1F** | NO |
|  | **4F** | Duodenum congestion |
|  | **5F** | NO |
|  | **6M** | NO |
|  | **9M** | NO |
|  | **10M** | NO |
| **Control** | **11F** | NO |
|  | **12F** | NO |
|  | **13F** | NO |
|  | **14F** | NO |
|  | **15F** | NO |
|  | **16M** | NO |
|  | **17M** | Pancreas with thick appearance |
|  | **18M** | NO |
|  | **19M** | Pancreas with thick appearance |
|  | **20M** | Presence of nodules and vesicles in the lung |

## Table S3: Weekly clinical evaluation of test and control groups (rabbits)

| **Group** | **Animal** | **Day** | **Day** | **Day** | **Day** | **Day** | **Day** | **Day** | **Day** | **Day** | **Day** | **Day** | **Day** | **Day** | **Day** | **Day** | **Day** | **Day** | **Day** | **Day** | **Day** | **Day** | **Day** | **Day** | **Day** | **Day** | **Day** |
| --- | --- | --- | --- | --- | --- | --- | --- | --- | --- | --- | --- | --- | --- | --- | --- | --- | --- | --- | --- | --- | --- | --- | --- | --- | --- | --- | --- |
|  |  | **0** | **7** | **14** | **21** | **28** | **35** | **42** | **49** | **56** | **63** | **70** | **77** | **84** | **91** | **98** | **105** | **112** | **119** | **126** | **133** | **140** | **147** | **154** | **161** | **168** | **175** |
| **Test** | **1F** | NO | NO | NO | NO | NO | 1* | --- | --- | --- | --- | --- | --- | --- | --- | --- | --- | --- | --- | --- | --- | --- | --- | --- | --- | --- | --- |
|  | **2F** | NO | NO | NO | NO | NO | NO | NO | NO | NO | NO | NO | NO | NO | NO | NO | NO | NO | NO | NO | NO | NO | NO | NO | NO | NO | NO |
|  | **3F** | NO | NO | 1* | --- | --- | --- | --- | --- | --- | --- | --- | --- | --- | --- | --- | --- | --- | --- | --- | --- | --- | --- | --- | --- | --- | --- |
|  | **4F** | NO | NO | NO | NO | NO | NO | NO | NO | NO | NO | NO | NO | NO | NO | NO | NO | NO | NO | NO | NO | NO | NO | NO | NO | NO | NO |
|  | **5F** | NO | NO | NO | NO | NO | NO | NO | 1* | --- | --- | --- | --- | --- | --- | --- | --- | --- | --- | --- | --- | --- | --- | --- | --- | --- | --- |
|  | **11F** | NO | NO | NO | NO | NO | NO | NO | NO | NO | NO | NO | NO | NO | NO | NO | NO | NO | NO | NO | NO | NO | NO | NO | NO | NO | NO |
|  | **6M** | NO | NO | NO | NO | NO | NO | NO | NO | NO | NO | NO | NO | NO | NO | NO | NO | NO | NO | NO | NO | NO | NO | NO | NO | NO | NO |
|  | **7M** | NO | NO | NO | NO | NO | NO | NO | NO | NO | NO | NO | NO | NO | NO | NO | NO | NO | NO | NO | NO | NO | NO | NO | NO | NO | NO |
|  | **8M** | NO | NO | NO | NO | NO | NO | NO | NO | NO | NO | NO | NO | NO | NO | NO | NO | NO | NO | NO | 1* | --- | --- | --- | --- | --- | --- |
|  | **9M** | NO | NO | NO | NO | NO | NO | NO | NO | NO | NO | NO | NO | NO | NO | NO | NO | NO | NO | NO | NO | NO | NO | NO | NO | NO | NO |
|  | **10M** | NO | NO | NO | NO | NO | NO | NO | NO | NO | NO | NO | NO | NO | NO | NO | NO | NO | NO | NO | NO | NO | NO | NO | NO | NO | NO |
| **Control** | **12F** | NO | NO | NO | NO | NO | NO | NO | NO | NO | NO | NO | NO | NO | NO | NO | NO | NO | NO | NO | NO | NO | NO | NO | NO | NO | NO |
|  | **13F** | NO | NO | NO | NO | NO | NO | NO | NO | NO | 1* | --- | --- | --- | --- | --- | --- | --- | --- | --- | --- | --- | --- | --- | --- | --- | --- |
|  | **14F** | NO | NO | NO | NO | NO | NO | NO | NO | NO | 1* | --- | --- | --- | --- | --- | --- | --- | --- | --- | --- | --- | --- | --- | --- | --- | --- |
|  | **15F** | NO | NO | NO | NO | NO | NO | NO | NO | NO | NO | NO | NO | NO | NO | NO | NO | NO | NO | NO | NO | NO | NO | NO | NO | NO | NO |
|  | **16F** | NO | NO | NO | 1* | --- | --- | --- | --- | --- | --- | --- | --- | --- | --- | --- | --- | --- | --- | --- | --- | --- | --- | --- | --- | --- | --- |
|  | **17M** | NO | NO | NO | NO | NO | NO | NO | NO | NO | NO | NO | NO | NO | NO | NO | NO | NO | NO | NO | NO | NO | NO | NO | NO | NO | NO |
|  | **18M** | NO | 11 | 1* | --- | --- | --- | --- | --- | --- | --- | --- | --- | --- | --- | --- | --- | --- | --- | --- | --- | --- | --- | --- | --- | --- | --- |
|  | **19M** | NO | NO | NO | NO | NO | NO | NO | NO | NO | NO | NO | NO | NO | NO | NO | NO | NO | 1■ | --- | --- | --- | --- | --- | --- | --- | --- |
|  | **20M** | NO | NO | NO | NO | NO | NO | NO | NO | NO | NO | NO | NO | NO | NO | NO | NO | NO | NO | NO | NO | NO | NO | NO | NO | NO | NO |
|  | **21M** | NO | NO | NO | NO | NO | NO | NO | NO | NO | NO | NO | NO | NO | NO | NO | 1▲ | --- | --- | --- | --- | --- | --- | --- | --- | --- | --- |
|  | **22M** | NO | NO | NO | NO | NO | NO | NO | NO | NO | NO | NO | NO | NO | NO | NO | NO | NO | NO | NO | NO | NO | NO | NO | NO | NO | NO |

F: female; M: male. Clinical alterations observed during the study ranged from no observations (NO) to various symptoms including death (1), convulsion (2), mutilation (3), prostration (4), ataxia (5), tremors (6), local inflammation (7), dyspnea (8), tearing (9), salivation (10), diarrhea (11), piloerection (12), and cachexia (13). * death by false route of administration. ^▲^ animal euthanized due to spinal injury; ^■^ animal euthanized due to facial abscess

## Table S4: Necropsy Findings of Test and Control Groups (Rabbits)

| **Group** | **Animal** | **Macroscopic findings** |
| --- | --- | --- |
| **Test** | **6M** | Dilatation of urinary bladder and strangulation on one border of spleen |
|  | **7M** | Hemorrhagic areas and abscess in the lung |
|  | **9M** | NO |
|  | **10M** | NO |
|  | **2F** | NO |
|  | **4F** | NO |
|  | **11F** | Edema and hemorrhagic areas in the lung |
| **Control** | **17M** | Congestion in cecum and duodenum, pancreas with dark color |
|  | **20M** | Congestion in cecum and duodenum |
|  | **22M** | NO |
|  | **12F** | Lymph node and pancreas with dark color |
|  | **15F** | Pancreas with dark color, left adrenal with small vesicle |

# **Table S5: Mean food intake (g) - rats**

| **Sex Group Month 1** | **Month 2** | **Month 3** | **Month 4** | **Month 5** | **Month 6** |
| --- | --- | --- | --- | --- | --- |
| **Test** 15.5 ± 1.7  **Females**  **Control** 15.4 ± 5.7 | 15.8 ± 1.3  12.3 ± 1.9 | 14.0 ± 1.5  15.8 ± 6.5 | 14.9 ± 1.9  14.0 ± 1.5 | 16.5 ± 3.9  15.3 ± 2.0 | 17.3 ± 2.8  11.2 ± 1.3 |
| **p value** 0,16* | *<0.001* | 0.48 | 0.34 | 0.49 | *<0.001* |
| **Test** 26.0 ± 5.3  **Males**  **Control** 26.9 ± 2.4 | 23.5 ± 2.1  25.8 ± 3.1 | 23.5 ± 2.8  25.0 ± 3.4 | 21.9 ± 6.3  26.8 ± 3.0 | 24.0 ± 10.5  28.0 ± 3.3 | 25.2 ± 4.5  26.0 ± 4.7 |
| **p value** 0,14* | 0.10* | 0.35 | 0.09 | 0.51 | 0.77 |

Data expressed as mean ± standard deviation; * Mann-Whitney test.

# **Table S6: Mean water intake (g) - rats**

| **Sex Group Month 1** | **Month 2** | **Month 3** | **Month 4** | **Month 5** | **Month 6** |
| --- | --- | --- | --- | --- | --- |
| **Test** 25.9 ± 2.8  **Females**  **Control** 23.3 ± 2.4 | 32.9 ± 5.3  26.9 ± 8.8 | 28.8 ± 8.3  25.8 ± 4.2 | 40.0 ± 19.5  24.4 ± 5.5 | 29.2 ± 5.7  26.1 ± 12.2 | 33.3 ± 10.0  24.2 ± 3.9 |
| **p value** 0,07 | 0.12 | 0.39 | *0.06** | 0.64 | 0.08 |
| **Test** 39.8 ± 5.8  **Males**  **Control** 45.8 ± 10.8 | 45.0 ± 6.8  43.3 ± 13.4 | 43.6 ± 8.5  47.1 ± 10.2 | 36.7 ± 4.7  46.1 ± 6.4 | 37.5 ± 4.2  41.3 ± 18.3 | 33.3 ± 6.7  51.9 ± 18.6 |
| **p value** 0,19* | 0.76 | 0.49 | *0.03* | 0.60 | 0.15 |

| **Sex Group Month 1** | **Month 2** | **Month 3** | **Month 4** | **Month 5** | **Month 6** |
| --- | --- | --- | --- | --- | --- |
| **Test** 25.9 ± 2.8  **Females**  **Control** 23.3 ± 2.4 | 32.9 ± 5.3  26.9 ± 8.8 | 28.8 ± 8.3  25.8 ± 4.2 | 40.0 ± 19.5  24.4 ± 5.5 | 29.2 ± 5.7  26.1 ± 12.2 | 33.3 ± 10.0  24.2 ± 3.9 |
| **p value** 0,07 | 0.12 | 0.39 | *0.06** | 0.64 | 0.08 |
| **Test** 39.8 ± 5.8  **Males**  **Control** 45.8 ± 10.8 | 45.0 ± 6.8  43.3 ± 13.4 | 43.6 ± 8.5  47.1 ± 10.2 | 36.7 ± 4.7  46.1 ± 6.4 | 37.5 ± 4.2  41.3 ± 18.3 | 33.3 ± 6.7  51.9 ± 18.6 |
| **p value** 0,19* | 0.76 | 0.49 | *0.03* | 0.60 | 0.15 |

Data expressed as mean ± standard deviation; * Mann-Whitney test.

# **Table S7: Mean food intake (g) - rabbits**

| **Group** | **Month 1** | **Month 2** | **Month 3** | **Month 4** | **Month 5** | **Month 6** |
| --- | --- | --- | --- | --- | --- | --- |
| **Test** | 166.3±34.4 | 172.0±60.0 | 135.5±52.0 | 118.8±43.6 | 187.4±29.3 | 157.1±49.0 |
| **Control** | 182.3±38.9 | 150.7±21.2 | 160.7±50.7 | 179.7±49.2 | 138.8±32.5 | 174.8±51.2 |
| **p value** | 0.40 | 0.38 | 0.38 | *0.02* | *0.02* | 0.56 |

Data expressed as mean ± standard deviation. * Mann-Whitney test.

# **Table S8: Mean water intake (g) - rabbits**

| **Group** | **Month 1** | **Month 2** | **Month 3** | **Month 4** | **Month 5** | **Month 6** |
| --- | --- | --- | --- | --- | --- | --- |
| **Test** | 298.8±166.9 | 286.3±177.4 | 248.8±158.5 | 232.9±158.5 | 328.3±70.5 | 305.7±116.9 |
| **Control** | 331.3±143.3 | 290.0±203.2 | 260.0±146.7 | 208.3±142.2 | 264.0±61.9 | 286.0±136.1 |
| **p value** | 0.68 | 0.80* | 0.89 | 0.78 | 0.15 | 0.79 |

Data expressed as mean ± standard deviation. * Mann-Whitney test.
